# Editorial: Enrichment of social skills in adolescent and young adults with high-functioning autism spectrum disorder

**DOI:** 10.3389/fpsyt.2022.994914

**Published:** 2022-08-08

**Authors:** Antonio Narzisi, Katherine K. M. Stavropoulos

**Affiliations:** ^1^IRCCS Stella Maris Foundation, Pisa, Italy; ^2^School of Education, University of California, Riverside, Riverside, CA, United States

**Keywords:** Autism Spectrum Disorder (ASD), social skills, adolescent, young adults, PEERS^®^

Among the core issues of Autism Spectrum Disorder (ASD), challenges with social skills (SS) are prominent (1). Indeed, adolescent and young adults with high functioning ASD exhibit difficulties in establishing friendships and playing cooperative games, and are consequently poorly included in social life and more vulnerable to demoralization, depression, anxiety, and low self-esteem (2).

Given that social participation is a key predictor of quality of life and overall functioning, it is crucial to enhance the SS of affected adolescents and young adults. Many interventions aimed at improving SS have been explored, but few of them are evidence-based and/or have reliable and valid methods for measuring outcomes (3).

In the Research Topic we edited, two articles utilized the PEERS® model (4). It is among the few evidence-based clinical interventions, with neuroscience outcomes (5), for adolescents and young adults with ASD. Over the years, the PEERS® model has spread internationally through rigorous cultural adaptation procedures.

In the article by Oh et al. the authors presented the Korean adaptation of the PEERS® for Young Adults (PEERS®-YA). They conducted an RCT with immediate treatment (TG) and delyed treatment (DTG) groups. Two important findings were discussed. First, results suggested that improved SS knowledge was related to reduced depression and anxiety symptoms. Second, results suggested that content of the Korean adaptation of PEERS® (PEERS®-YA-K) is acceptable to Koren culture and feasible to implement.

The other published article concerning the PEERS® model, this time with autistic adolescents, is that of McLeod and McCrimmon. This article studied how age, gender, emotional intelligence, intellectual ability, and/or autism symptomatology could predict improvement in SS after the PEERS® intervention. Authors reported that only intellectual ability, specifically perceptual reasoning, significantly predicted SS outcomes, suggesting that autistic youth with specific cognitive profiles may benefit more from PEERS®.

Difficulties in SS are a serious issues among children with ASD and often at school the opportunities for peer interaction and to practice SS may be infrequent. The exploratory article by McDaniel et al. highlights the need to implement extracurricular clubs for building social competence, as it is clear that high schools are failing to meet the unique needs of students with ASD. A focus on building SS is crucial to prepare these young people for productive and fulfilling lives post-high school. Peer-mediated instruction and intervention may exist in some high schools, but students with ASD who spend most of their time in general education may not have access to these programs. Due to complex diploma and graduation requirements, high school students' schedules are often too full to make time for SS interventions, and SS interventions are not typically part of the curriculum for diploma-bound students with ASD. For this reason, extracurricular clubs and activities may serve to address these unmet social needs. For example, participation in extracurricular clubs is one of the recommended activities in the school-based PEERS® curriculum due to the typical opportunities to engage in peer interaction (4).

The results of McDaniel et al. suggest several recommendations for school personnel in high schools. It is important that school sites consider the opportunities that all students, and particularly those with disabilities, have to explore and learn about extracurricular opportunities on campus. This opportunity is ultimately an issue of equity and access. Special education departments should ensure that their staff are well-versed in the school's club offerings so that special educators can confidently discuss club opportunities with students with autism and their parents.

The difficulties in the area of social communication of persons with ASD were highlighted even more during the COVID pandemic (6). In this sense, Kumazaki et al. developed a communication training system using a tele-operated robot; in this system, a PC and a robot was prepared for each participant. The participants were grouped in pairs and communicated with each other through the tele-operated robot. The aim of this study was to test whether this system can maintain motivation for training in individuals with ASD and whether the system was useful for improving communication skills. Participants were randomly assigned to one of two groups: taking a class by teachers alone (TCT) group or robot-mediated communication exercise (RMC) group. Participants in the TCT group took a class about communication skills from their teacher. Participants in the RMC group, in addition to taking a class by teacher, were grouped in pairs and communicated with each other through the tele-operated robot once a week over 4 weeks (for a total of five sessions). Overall, this study revealed that this system was useful for improving communication skills (e.g., listening to the thoughts or feelings of others). Teaching communication skills under COVID pandemic conditions is important, and this study demonstrated the feasibility of communication training using tele-operated robots.

Finally, the article presented by Keller et al. described a narrative experience of real-life—social and motor—skills training in adults with ASD. The authors developed a project addressing social, physical, and mental health difficulties in real-life walking down the Francigena route for 9 days with 12 autistic people. The project involved, (1) implementing daily sessions of social skills training which were designed to be immediately generalized and used throughout the day; (2) educational movement and walking activity programs were led by a fitness coach; (3) the creation of walking peers' social community with a strong and relevant impact on adults with ASD social life respecting every person's individuality; (4) provision of social reinforcers to reduce the stigma of people with ASD and the experienced perception of low self-esteem, especially related to bullying.

The articles in this Research Topic offered interesting insights on the need to work on the implementation of SS in clinical practice as well as in school, extracurricular and everyday life settings.

## Author contributions

All authors listed have made a substantial, direct, and intellectual contribution to the work and approved it for publication.

## Funding

AN has been partially supported by grant-RC 2.06 and the 5 × 1000 voluntary contributions, Italian Ministry of Health.

## Conflict of interest

Author AN was employed by IRCCS Stella Maris Foundation. The remaining author declares that the research was conducted in the absence of any commercial or financial relationships that could be construed as a potential conflict of interest.

## Publisher's note

All claims expressed in this article are solely those of the authors and do not necessarily represent those of their affiliated organizations, or those of the publisher, the editors and the reviewers. Any product that may be evaluated in this article, or claim that may be made by its manufacturer, is not guaranteed or endorsed by the publisher.
